# Monitoring Muscle-Tendon Adaptation Over Several Years of Athletic Training and Competition in Elite Track and Field Jumpers

**DOI:** 10.3389/fphys.2020.607544

**Published:** 2020-12-16

**Authors:** Kiros Karamanidis, Gaspar Epro

**Affiliations:** Sport and Exercise Science Research Centre, School of Applied Sciences, London South Bank University, London, United Kingdom

**Keywords:** Achilles tendon, triceps surae, time course, imbalance, athletic training, tendinopathy

## Abstract

Differences in muscle and tendon responsiveness to mechanical stimuli and time courses of adaptive changes may disrupt the interaction of the musculotendinous unit (MTU), increasing the risk for overuse injuries. We monitored training-induced alterations in muscle and tendon biomechanical properties in elite jumpers over 4 years of athletic training to detect potential non-synchronized adaptations within the triceps surae MTU. A combined cross-sectional and longitudinal investigation over 4 years was conducted by analyzing triceps surae MTU mechanical properties in both legs via dynamometry and ultrasonography in 67 elite track and field jumpers and 24 age-matched controls. Fluctuations in muscle and tendon adaptive changes over time were quantified by calculating individual residuals. The cosine similarity of the relative changes of muscle strength and tendon stiffness between sessions served as a measure of uniformity of adaptive changes. Our cross-sectional study was unable to detect clear non-concurrent differences in muscle strength and tendon stiffness in elite jumpers. However, when considering the longitudinal data over several years of training most of the jumpers demonstrated greater fluctuations in muscle strength and tendon stiffness and hence tendon strain compared to controls, irrespective of training period (preparation vs. competition). Moreover, two monitored athletes with chronic Achilles tendinopathy showed in their affected limb lower uniformity in MTU adaptation as well as higher fluctuations in tendon strain over time. Habitual mechanical loading can affect the MTU uniformity in elite jumpers, leading to increased mechanical demand on the tendon over an athletic season and potentially increased risk for overuse injuries.

## Introduction

Tendons are an integral part of musculotendinous units (MTUs) and their primary function is to transmit forces from muscles to rigid bone levers to produce joint motion. As a part of their viscoelastic nature, elasticity of the tendons can enhance force and power generating capacity of MTUs by storing and releasing strain energy and by allowing muscles to operate under more favorable conditions related to the force-length-velocity relationship (Biewener and Roberts, 2000; Hof et al., 2002). Thus the mechanical properties of tendons need to be considered as important determinants of musculoskeletal function, and it is not surprising that they have been shown to affect athletic performance (Bojsen-Møller et al., 2005; Stafilidis and Arampatzis, 2007). Not only are tendons vitally important for movement and sports performance, movement and exercise are also essential for tendons. As living tissue, tendons respond to mechanical loading by changing their metabolism, which can lead to alterations in their material composition (e.g., changes in extra-cellular matrix or cross-linking) and their morphological properties (cross-sectional area) with the latter as a more long-term response (Bohm et al., 2015a; Wiesinger et al., 2015). Accordingly, when a MTU is constantly experiencing increased mechanical loading (e.g., by resistance training) it is generally observed that the associated adaptive enhancements in muscle strength lead also to a markedly greater tendon stiffness (Kubo et al., 2001, 2012; Arampatzis et al., 2007a,b; Bohm et al., 2014). The reported modifications in tendon stiffness may exhibit a protective homeostatic process to withstand greater functional demand due to muscle strength gains. Nevertheless, the muscle strength gains seem to be merely in a moderate association with modifications in tendon mechanical properties (Arampatzis et al., 2007a,b). This may be explained by discrepancies between muscle and tendon in their adaptive response to the experienced mechanical stimuli (Arampatzis et al., 2007a) and in the timescale of adaptation (Kubo et al., 2012) with distinctively slower response rates for tendons due to lower tissue turnover (Heinemeier et al., 2013). Hence, an imbalance between the generated muscle forces and the strength of the tendon may occur over the course of training process, placing the tendon under greater mechanical demand (i.e., higher strain).

Compared to tendons, muscles seem to possess superior adaptability in reaction to a broad variety of exercise modalities (Moss et al., 1997), the former responding most effectively to mechanical loads creating high magnitudes of tendon strain over extended time durations (Arampatzis et al., 2007a; Bohm et al., 2014). These adaptive discrepancies within the MTU could become crucial for athletes constantly involved with high mechanical loading profiles in their training process, particularly in sport disciplines with frequent plyometric loading, such as track and field sprinters and jumpers (Stefanyshyn and Nigg, 1997, 1998; Dorn et al., 2012; Willwacher et al., 2017). By its nature plyometric training is characterized by high magnitude mechanical loading, though the time spans over which those high forces are applied tend to be rather short. This might be a reason why various plyometric loading regimens do not always lead to clear adaptive increases in tendon stiffness along with muscle strength gains (Kubo et al., 2007; Fouré et al., 2010; Houghton et al., 2013; Bohm et al., 2014; Hirayama et al., 2017). From a biomechanical viewpoint, muscle strength improvements with insufficient compensatory adaptations in tendon mechanical properties would potentially increase the mechanical demand for the tendon (i.e., higher strain). In fact, it has recently been demonstrated that habitual athletic training that consists mainly of plyometric loading affects the uniformity of muscle and tendon adaptation in adolescents (Mersmann et al., 2016) with potential implications for injury and tendinopathy (Wren et al., 2003; Fredberg and Stengaard-Pedersen, 2008). The fact that athletic training for elite athletes consists of complex loading variations and includes different training modalities (Haugen et al., 2019) could lead to considerable fluctuations in muscle strength in the intermediate term, which may have implications not only for the performance but also for the integrity of the tendon. Regarding that a clear pathogenesis of the development of tendon overuse is still unknown, the above mentioned provides a more mechanistic explanation along other more physiological concepts behind pathological changes in tendon (e.g., immunoregulation/excessive immune response).

The Achilles tendon (AT) is susceptible to injury potentially due its noticeably low safety factor (the ratio of ultimate and operational stress) when compared with other tendons (Ker et al., 1988; Magnusson et al., 2001). Accordingly it does not seem so unexpected that there is a high prevalence of AT tendinopathies in adult athletes (Janssen et al., 2018). This supports the assumption that periods of asynchronous muscle-tendon adaptation in elite jumping athletes might be of clinical relevance and that further research is needed to explore whether athletic training is an influential factor in non-uniform adaptation between muscle and tendon. To our knowledge the time course of muscle and tendon adaptations in adult athletes over several years of athletic training and competition is unknown.

The aim of the current investigation was to regularly monitor training-induced alterations in triceps surae (TS) muscle strength, tendon stiffness and tendon strain in the legs of elite jumpers over several years of training in order to detect potential non-uniformities within the TS MTU due to habitual mechanical loading. Potential alterations in TS MTU properties in elite athletes were compared with the variations in a control group of healthy recreationally active adults of similar age range. On the basis that elite athletes are subjected to high variation in mechanical loading profiles over a season, we hypothesized that elite athletes experience greater fluctuations in MTU mechanical properties over time and demonstrate temporary increases in mechanical demand on the tendon as a result of non-uniform MTU adaptation. For this reason we conducted a combined cross-sectional and longitudinal investigation by analyzing a total of 67 healthy young elite jump track and field athletes competing at international level, from which a sub-group of 18 elite jumpers were monitored regularly over 1 year of training. As part of this long-term investigation six of the elite athletes were measured regularly over a 4-year period to investigate possible differences between the preparation and competition periods. Two additional elite athletes with unilateral AT tendinopathy and one athlete following unilateral AT reconstruction will be discussed separately.

## Materials and Methods

### Participants and Experimental Design

In this investigation on TS MTU adaptation we were able to voluntarily recruit 67 healthy young elite international level jumping event track and field athletes (35 male and 32 female) from the German national team (age: 23 ± 4 years; body mass: 73 ± 11 kg; body height: 183 ± 9 cm; mean and standard deviation; for further description and personal records see Supplementary Material). Additionally, 24 young healthy recreationally active (12 male and 12 female; age: 24 ± 3 years; body mass: 72 ± 12 kg; body height: 177 ± 10 cm) acted as control group. Excluded were athletes with prior AT injuries (ruptures, tendinopathy etc.) during the preceding six months, as it may have possibly affected the findings. Accordingly in total 91 subjects were included in our cross-sectional investigation. Next to the healthy participants, three additional athletes with current AT injuries (two athletes with chronic tendinopathy and one with a recent tendon rupture) voluntarily participated in the study and were separately included to the study. The study was ethically approved by the local committee of the German Sport University Cologne and all participants provided their written informed consent prior to the study in accordance to the recommendations in the Declaration of Helsinki.

**Figure 1:**
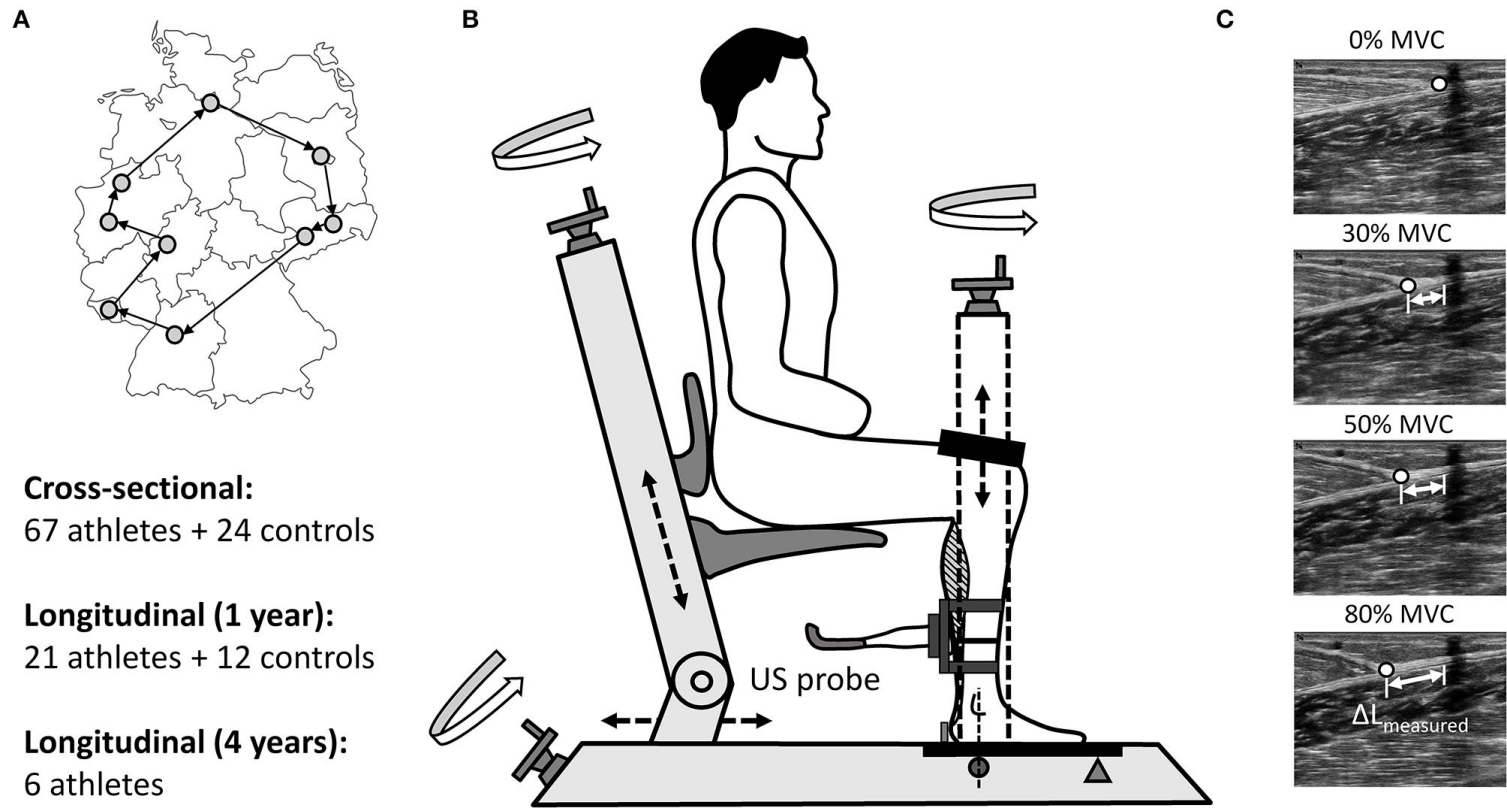
Measurement locations at the Olympic training centers in Germany incl. study design **(A)** and the used custom-made analysis device (TEMULAB®, Protendon GmbH & Co. KG, Aachen, Germany) to assess TS MTU mechanical properties incorporating dynamometry and ultrasonography **(B)**. In order to determine the force-elongation relationship of the tendon the displacement of the myotendinous junction (MTJ) of the m. gastrocnemius medialis (ΔL_measured_) was digitized at rest (0%) and at three sustained contractions (target joint moment constantly held for 3s) at fixed target ankle joint moment levels (30, 50, and 80%; **C**).

As a part of the day-to-day training routine at their corresponding Olympic training centers or training camps (Figure 1A), the TS MTU biomechanical properties (maximal ankle plantar flexion moment, AT stiffness and maximal tendon strain) were assessed in both legs of all participating athletes, concurrently or precisely prior to the competition period (cross-sectional investigation). In order to distinguish between the limbs, the preferred jumping leg was considered as the take-off leg and the contralateral leg as the swing leg. The hop leg in triple jumpers was defined as the take-off leg. Similar to the athletes, measurements with the control group were performed on both limbs in the cross-sectional investigation (take-off leg defined via a questionnaire as the preferred jumping leg), whereas in the longitudinal investigation only the take-off leg was analyzed. In the injured athletes, the comparison was made between the healthy and affected (injured) limb.

### Longitudinal Investigation Over 1 Year

Due to the a lack of appropriate longitudinal data of MTU adaptive changes in elite track and field athletes a power analysis for a target sample size was not possible, therefore it was aimed to recruit as many participants as possible. Out of the 91 healthy participants from our cross-sectional investigation, we were able to recruit 40 elite athletes and 15 controls to participate over a minimum of 1 year observation period. However, only 18 athletes and 12 controls were considered as valid for our longitudinal investigation over 1 year by reaching our criteria for the statistical analysis (set criteria: regular measurement about every three- to five-weeks) while the rest dropped out due to missing time, various injuries (including lower limb MTU injuries) or due to other reasons. Next to the 18 monitored healthy athletes we were able to recruit and regularly measure two elite athletes with a chronic unilateral AT tendinopathy and one elite jumping athlete 10 months after AT rupture and reconstruction monitored over a follow up time period of 2 years. The data of these three athletes with AT injury were not included in the statistical analysis of the pool of healthy elite athletes and were considered separately. For the statistical analysis of the elite athletes (*n* = 21), measurements were conducted on both limbs at every three- to five-weeks over the 1 year observation window (only for the elite athlete with AT rupture less frequent measurements were conducted). For the control group only the take-off leg was considered, as previous studies have shown similar TS MTU mechanical properties between limbs in healthy adults (Bohm et al., 2015b) and thereby to reduce the amount of measurements taken.

### Longitudinal Investigation Over 4 Years

Twelve jumpers from the initial pool of healthy elite athletes agreed to participate over an extended 4 year time period of TS MTU monitoring. However, only in six elite athletes it was possible to determine the bilateral MTU biomechanical properties about every three- to five-weeks over the 4 years period, therefore all other athletes were excluded from further analysis (dropped out due to missing time, injury or other reasons). Those regularly monitored six elite athletes served for the investigation of differences between the preparation and competition periods in bilateral MTU adaptive changes over 4 years.

### Analysis of Muscle and Tendon Mechanical Properties

The TS MTU biomechanical properties (maximal ankle plantar flexion moment, AT stiffness and maximal AT strain) were assessed in all participants on a custom-made device (Figure 1B) using synchronous ultrasonography and dynamometry as in our previous study (Epro et al., 2019). Briefly, the participants were positioned with their knee and ankle joints fixed at 90° (with their foot and thigh perpendicular to the shank) and their foot set on a strain-gauge type dynamometer (1000Hz; TEMULAB®, Protendon GmbH & Co. KG, Aachen, Germany; Figure 1B). The foot was set on the dynamometer (Figure 1B) with the midpoint of the malleolus lateralis aligned with the dynamometer's axis of rotation (Ackermans et al., 2016). A laser-guided electrical potentiometer system measuring the linear displacement was used to position each participant in a standardized manner, which allowed recreating the exact position for each individual at any measurement time point. Prior to each measurement, all participants performed an individual warm-up, which was succeeded by a standardized visually guided warm-up in the software consisting of several submaximal and three maximal isometric contractions to “precondition” the tendon (Maganaris, 2003).

Subsequently, the maximal ankle plantar flexion moment as well as the force–elongation relationship of the tendon during the loading phase were assessed by performing isometric ankle plantar flexion contractions at different joint moment levels: three verbally encouraged maximal voluntary ankle plantar flexion contractions (MVC), succeeded by three visually guided sustained contractions at 30, 50, and 80% of the maximal joint moment obtained in the prior MVCs. The resultant joint moments acting about the ankle joint were calculated using inverse dynamics and a preceding passive measurement (relaxed muscles in the fixed position) was performed to account for the gravitational moments. The alignment of the axes of rotation of the ankle and the dynamometer (Figure 1B) allowed to consider the ankle joint moment to be quasi-equal with the acquired moment of the force plate (Ackermans et al., 2016). Nevertheless, it needs to be noted, that the resultant ankle plantar flexion moment is an approximation of the net moment generated by the TS muscle, as the current setup did not account for the individual moment contributions of the synergistic agonist muscles and the antagonist dorsiflexors. Subsequently, the resultant ankle joint moment was further used to calculate the acting AT force by dividing it by the tendon moment arm acquired from a previous study (Maganaris et al., 1998). In order to exclude the effect of any potential changes in body mass on the calculations of fluctuations in muscle strength over time, we used the absolute maximal joint moments (Nm) instead of body mass normalized moments.

After tendon preconditioning, the AT resting length was measured using the integrated laser-guided electrical potentiometer system as the distance from the myotendinous junction (MTJ) of the m. gastrocnemius medialis to the most proximal point of the tuber calcanei (both defined and marked beforehand using ultrasonography). Similar to the positioning of each participant on the dynamometer, the laser-guided electrical potentiometer system allowed maintaining the probe position constant for each subject and leg during all measurements. During the following plantar flexion contractions the movement of the MTJ of the m. gastrocnemius medialis was analyzed with a firmly fixed 40 mm linear array ultrasound probe (27 Hz; MyLab™One, Esaote; Genoa, Italy) and manual digitization using a custom-made software in Python (ver. 2.7.0; Figure 1C). The ultrasound probe was secured above the MTJ on the shank using a casing with adjustable straps to avoid movement in relation to the skin. The MTJ displacement was traced at the resting state (0% MVC) and in the following three sustained contractions (steadily held target joint moment for 3 s) at the target ankle joint moments (30, 50, 80% MVC; Figure 1C). The participants had to repeat the specific trial, when they were unsuccessful to hold a steady state for 3s within a range of ±5% of the target joint moment. A linear extrapolation of the elongation at 50 and 80% target joint moments was used to estimate the tendon elongation at maximal ankle joint moment (100%) as in Ackermans et al. (2016), because specific strict instructions about loading rate and maintaining the generated moments at certain given level may limit the ability to contract maximally. Moreover, this approach to calculate maximal tendon strain can be considered as a valid measure as tendon elongation between 50 and 100% of the MVC can be assumed to be rather linear and because a considerable amount of the net elongation occurs within the first 25% of the MVC (McCrum et al., 2018). The effect of the inevitable ankle joint angular rotation on the measured tendon elongation (Muramatsu et al., 2001) was taken into account during each contraction by subtracting the elongation caused by ankle joint changes. Ankle joint angle during each contraction was obtained through the inverse tangent of the ratio between the heel lift (measured via a heel-potentiometer) and the distance from the ankle joint axis to the fifth metatarsal bone (Ackermans et al., 2016). Subsequently, the ratio between the change in the calculated tendon force and the resultant tendon elongation between 30 and 80% of maximum tendon force was used to estimate the AT stiffness. Before conducting the current study we performed several pilot studies to test the accuracy and validity of the implemented method to assess the TS MTU mechanical properties. The main findings of these pilot studies are provided in the Supplementary Material under the chapter “Methodological considerations and pilot data.”

### Statistics

The normality of distribution and homogeneity of variance of the data was confirmed using the Shapiro-Wilk and Levene's test. For the cross-sectional analysis, a two-way repeated measures analysis of variance (ANOVA) was performed with the group (athletes vs. control) and leg (take-off vs. swing leg) as factors to investigate potential differences in TS muscle strength, AT stiffness and maximal AT strain. For the longitudinal investigations, firstly the uniformity of muscle and tendon adaptive changes over the 1 year time period in both elite athletes and control subjects were analyzed using individual absolute residuals (averaged over all measurement sessions) from a general linear model as a measure of fluctuations in TS MTU properties and secondly by forming cosine similarity (CS) of the relative changes between the individual measurement sessions (Mersmann et al., 2016):

$$\begin{array}{l} CS=\cos\left(\theta \right)=\;\frac{X\cdot Y}{\left||X||\cdot ||Y|\right|}\;=\;\frac{\sum_{i=1}^{n-1}X_{i}Y_{i}}{\sqrt{\sum_{i=1}^{n-1}X_{i}^{2}}\sqrt{\sum_{i=1}^{n-1}Y_{i}^{2}}} \end{array}$$

X and Y denote the vectors of the relative changes between sessions (*i* = 1,…, *n*) and *n* refers to the individual total number of measurements. CS values range between 1 (illustrating equal direction of the vectors, i.e., high adaptation similarity) to−1 (opposing direction, i.e., low adaptation similarity). For the 4 years investigation (eight consecutive seasons), the residuals and CS were averaged separately for the preparation (mid-March to mid-May and start of October to end of December) and competition periods (start of January to mid-March and mid of May to mid-September). A one-way ANOVA was implemented to investigate potential subject group-related differences (take-off leg vs. swing leg of athletes vs. take-off leg controls) in the fluctuations (residuals) and CS of muscle and tendon biomechanical properties over the 1 year time period. Potential training period (preparation vs. competition period) influence in the fluctuations and CS in MTU adaptation over 4 years of athletic training was examined using a two-way ANOVA with leg and training period as factors. In the case of a detected significant main effect or interaction a Bonferroni *post hoc* comparison was performed. In addition, in order to evaluate the strength of potential training-induced alterations in cross-sectional and longitudinal investigations the partial eta squared ($\eta_{\text{p}}^{\;2}$) was calculated (Cohen, 2013). Potential subject group differences in age, body mass and body height between elite athletes and controls were compared using an independent samples *t*-test. All statistical calculations and analyses were performed using custom-made MATLAB scripts (version 2020b; The MathWorks, Natick, MA, USA) or SPSS statistics software (version 26.0; IBM, Armonk, NY, USA) with the results in the text and figures provided either as means and standard deviation (SD) or as boxplots (median and interquartile range between 25th and 75th percentile along with minimum and maximum values) and the level of significance set at α = 0.05.

## Results

### Cross-Sectional Investigation: Elite Athletes vs. Controls

For the cross-sectional investigation 67 healthy elite jumpers (35 male and 32 female) and 24 recreationally active young adults (12 male and 12 female; control group) were considered for statistical analysis with respect to potential leg and subject group effects on MTU properties. Irrespective of the analyzed limb (take-off vs. swing), elite jumpers demonstrated in comparison to the control group significantly higher TS muscle strength (average values of both legs: athletes 303 ± 85 vs. controls 241 ± 65 Nm; subject group effect: F_(1, 89)_ = 10.415, *P* = 0.002, $\eta_{\text{p}}^{\;2}$ = 0.105) as well as greater tendon stiffness (653 ± 187 vs. 570 ± 131 N/mm; F_(1, 89)_ = 4.004, *P* = 0.048, $\eta_{\text{p}}^{\;2}$ = 0.043) with similar relative difference between groups (~20% in TS muscle strength vs. ~15% in tendon stiffness). Hence, maximal tendon strain was not significantly different between groups independent of the analyzed leg (average values of both legs: athletes 4.8 ± 1.1 vs. controls 4.8 ± 1.0%). Concerning the between leg analysis, we found limb-specific differences in TS MTU mechanical properties in jumpers but not in controls. Elite jumpers showed significantly higher muscle strength (310 ± 89 vs. 296 ± 84 Nm, *P* = 0.001, $\eta_{\text{p}}^{\;2}$ = 0.127) and tendon stiffness (675 ± 195 vs. 630 ± 186 N/mm, *P* < 0.001, $\eta_{\text{p}}^{\;2}$ = 0.226) in their take-off in comparison to their swing leg. No significant leg-specific differences were detected for maximal AT strain in the elite jumpers (take-off leg 4.6 ± 1.0 vs. swing leg 4.8 ± 1.1%). A significant group-difference between the elite jumpers and control group was found for the body height (183 ± 9 vs. 177 ± 10 cm for elite jumpers and control respectively, *P* = 0.008), but not for age (23 ± 4 vs. 24 ± 3 years) and body mass (73 ± 11 vs. 72 ± 12 kg).

### TS MTU Adaptive Changes Over 1 Year: Elite Athletes vs. Controls

**Figure 2:**
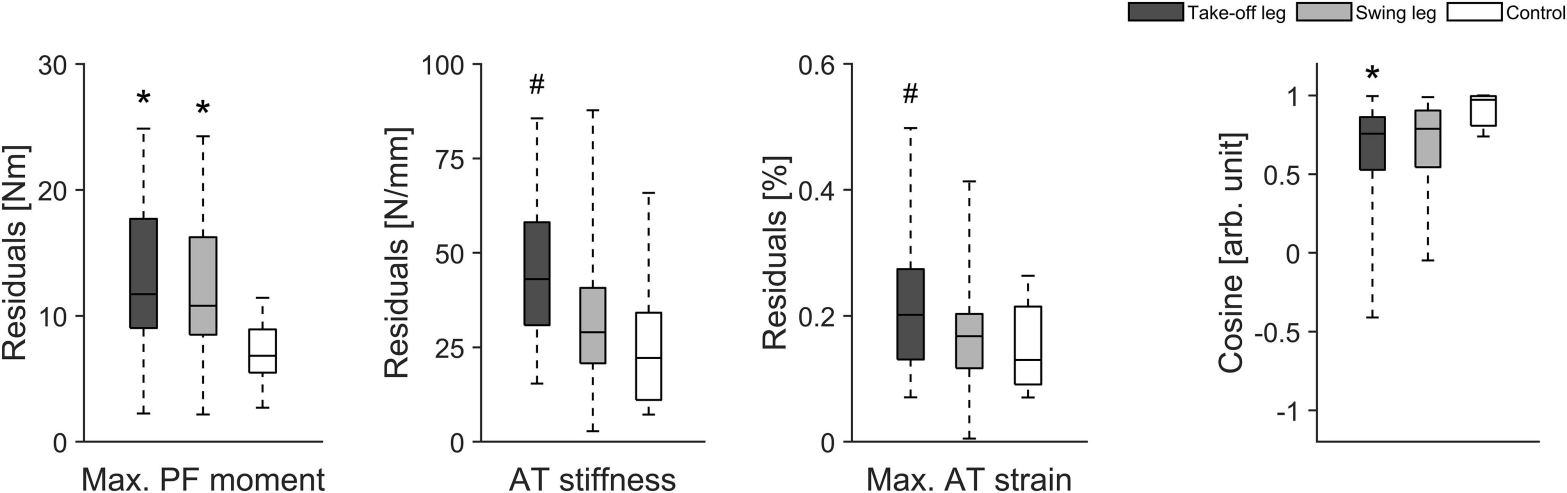
Box plots of muscle strength (Max. PF moment), tendon stiffness (AT stiffness) and maximal tendon strain (Max. AT strain) as well as the cosine similarity (Cosine) of the relative changes of muscle strength and tendon stiffness between sessions over the 1 year observation period in 18 healthy elite jumpers (take-off and swing leg) and 12 healthy control subjects (take-off leg). *Statistically significant difference to control (*P* < 0.05). ^#^Tendency for significant difference to control (0.05 < *p* < 0.1).

Eighteen healthy athletes (8 males and 10 females) were considered as valid for our longitudinal investigation over 1 year as they reached our criteria for the longitudinal statistical analysis (set criteria: measurement about every three- to five-weeks). When considering all data points analyzed within the 12 months observation period we found greater fluctuations of muscle strength over time in elite jumpers compared with controls irrespective of the analyzed leg (i.e., greater residuals; take-off leg of athletes vs. take-off leg control *P* = 0.010 and swing leg of athletes vs. take-off leg control *P* = 0.049; Figure 2). Furthermore, although not reaching statistical significance, there were tendencies for greater fluctuation in tendon stiffness (*P* = 0.074; Figure 2) and in maximal tendon strain for elite athletes take-off leg in comparison to control (*P* = 0.092; Figure 2). In addition, for the CS a main effect was detected (*P* = 0.030), with the athletes take-off leg in comparison to the control group demonstrated statistically significant (*P* = 0.026) lower similarities (lower CS) in their adaptive changes of muscle strength and tendon stiffness (Figure 2).

### TS MTU Adaptive Changes Over 4 Years: Preparation vs. Competition Period

**Figure 3:**
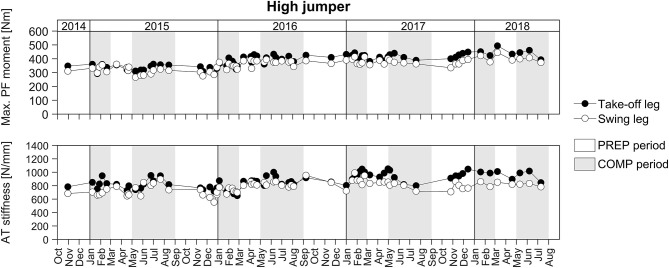
Adaptive changes in TS muscle strength (Max. PF moment) and tendon stiffness (AT stiffness) in the take-off and swing leg of one elite high jumper over a period of 4 years of athletic training separated into preparation (PREP) and competition (COMP) periods.

**Figure 4:**
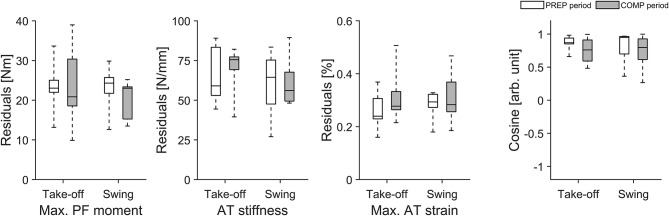
Box plots of residuals in muscle strength (Max. PF moment), tendon stiffness (AT stiffness) and maximal tendon strain (Max. AT strain) as well as in cosine similarity (Cosine) of the relative changes of muscle strength and tendon stiffness between sessions over 4 years during preparation (PREP) and competition (COMP) period in six elite jumpers (take-off and swing leg).

For the longitudinal investigation over 4 years we were able to analyse six healthy male elite athletes on a regular basis (about every three- to five-weeks) over eight preparation and eight competition periods. Exemplarily Figure 3 represents the changes in TS muscle strength and tendon stiffness in both legs of one elite high jumper over a period of 4 years of athletic training separated into preparation and competition periods. The total number of measurements for those six elite athletes over the 4 years period ranged from 36 to 73 with 51% measurements in the preparation and 49% measurements in the competition period. Independent of the analyzed leg, there were no significant differences in fluctuations of muscle strength, tendon stiffness and maximal tendon strain over time (no differences in the residuals) between preparation and competition period (Figure 4). Further, there were no significant differences in CS in the adaptive changes of muscle strength and tendon stiffness for the preparation in comparison to the competition period (Figure 4).

### TS MTU Adaptive Changes in Elite Athletes With AT Injury

**Figure 5:**
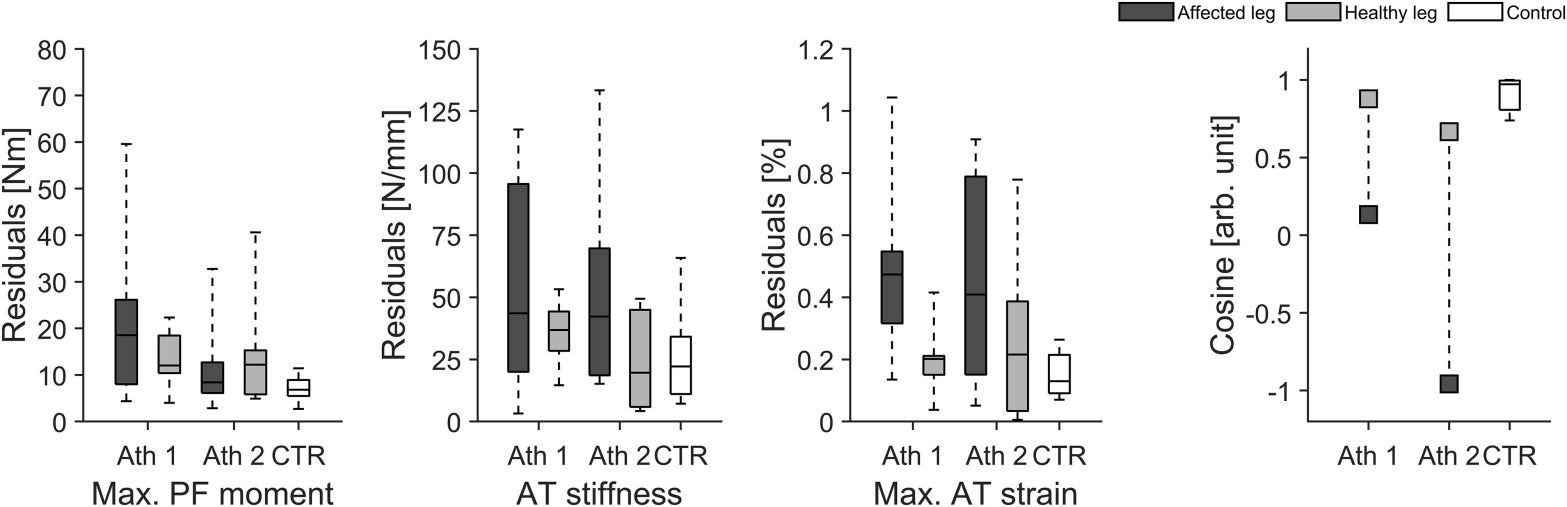
Box plots of residuals in muscle strength (Max. PF moment), tendon stiffness (AT stiffness) and maximal tendon strain (Max. AT strain) as well as the cosine similarity (Cosine) of the relative changes of muscle strength and tendon stiffness between sessions over the 1 year observation period for the affected and healthy leg in two elite jumpers (Ath 1 and Ath 2) with chronic AT tendinopathy and in the control group (CTR; take-off leg; *n* = 12).

In addition to the pool of 18 healthy athletes we were able to monitor two elite athletes with a unilateral AT tendinopathy and one elite athlete following a unilateral AT reconstruction over several years of athletic training. At baseline, the two elite athletes with a unilateral AT tendinopathy showed lower tendon stiffness in their affected (injured) limb compared to their unaffected limb (average values for both athletes: affected limb 564 ± 127 N/mm vs. healthy limb 690 ± 242 N/mm) with similar muscle strength values between limbs (303 ± 61 Nm vs. 312 ± 59 Nm). As a consequence, maximal tendon strain was for both athletes higher in their affected limb (5.3 ± 0.1 vs. 4.6 ± 0.2%). We were able to monitor these two elite athletes over a period of 1 year of athletic training and the two athletes with AT tendinopathy showed in their affected limb a lower uniformity (i.e., lower CS) in the adaptive changes of muscle strength and tendon stiffness in comparison to the healthy limb and healthy control group (Figure 5). Furthermore, the affected limb showed greater fluctuations (higher residuals) in muscle strength as well as in tendon stiffness and as a consequence in tendon strain over time in comparison to their healthy limb and healthy controls (Figure 5).

**Figure 6:**
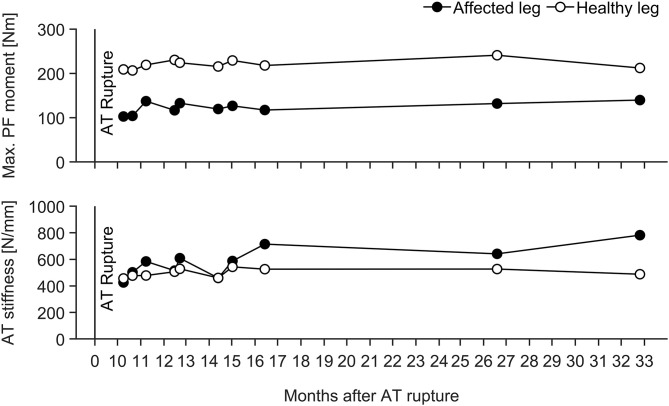
Adaptive changes in TS muscle strength (Max. PF moment) and tendon stiffness (AT stiffness) in the affected and healthy leg of an elite athlete with an Achilles tendon rupture and reconstruction during a follow up time period of 2 years. Maximal tendon strain ranged from 1.5 to 2.5% and from 3.9 to 4.9% for the affected and healthy leg over the entire observation window, respectively.

The remaining injured elite jumper was measured 10 months after an Achilles tendon rupture and reconstruction and we were able to monitor the jumper's bilateral MTU adaptive changes over a follow up time period of 2 years. The data in Figure 6 indicates that the affected leg shows on average lower muscle strength (~57%) but about 15% higher Achilles tendon stiffness (with more than 60% lower maximal strain) in comparison to the healthy leg despite over 2.5 years of intense rehabilitation and athletics training.

## Discussion

Muscles and tendons both adapt to mechanical loading. However, differences in their responsiveness to mechanical stimuli and the rates of adaptive changes may disrupt the fine-tuned interaction of the MTU, increasing the risk for overuse injuries. In the current work we monitored training-induced alterations in muscle strength and tendon stiffness in young elite jumpers over 4 years of athletic training in order to detect potential non-uniformities within the TS MTU due to habitual mechanical loading. When considering the longitudinal data over several years of athletic training most of the jumpers demonstrated greater fluctuations in TS muscle strength and tendon stiffness (and hence tendon strain) compared to controls, irrespective of training period (preparation vs. competition). Moreover, two elite athletes with chronic AT tendinopathy showed lower uniformity (lower CS) in their affected leg in the adaptive changes of muscle strength and tendon stiffness and higher fluctuations in tendon strain. These results support our hypothesis that habitual mechanical loading can affect the uniformity within the MTU in elite jumpers, suggesting a temporarily increased mechanical demand on the tendon over a season and potentially higher injury risk.

Although most resistance exercise interventions implementing high tendon strain magnitudes are regularly reporting comparable changes in tendon and muscle biomechanical properties (Kubo et al., 2001; Arampatzis et al., 2007a), purely plyometric exercise interventions show tendon adaptations that are not concurrent with those of skeletal muscle (Kubo et al., 2007, 2017; Fouré et al., 2010; Sáez-Sáez de Villarreal et al., 2010; Houghton et al., 2013; Bohm et al., 2014; Hirayama et al., 2017). A feasible explanation for this may be that tendons adapt less effectively to loading profiles characterized by short tendon strain durations (Bohm et al., 2014)—as in plyometric activities such as jumping or sprint running. Yet, our cross-sectional investigation comprising a total of 91 subjects did not detect clear non-concurrent increases in muscle strength and tendon stiffness in elite jumpers. Irrespective of the analyzed leg, differences were detected for both TS muscle strength and tendon stiffness (0.043 ≤ $\eta_{\text{p}}^{\;2}$ ≤ 0.105), and hence similar levels of strain during maximum contractions between athletes and controls were observed (on average 4.8% in both the athlete and the control group). Given that tendon strain was not significantly greater in athletes compared to controls, one might suggest that repetitive plyometric and habitual mechanical loading during training seems not to provoke any measurable non-uniform adaptation within the TS MTU in elite jumpers. Hence, it is reasonable to suggest that the reported alterations in tendon stiffness in elite athletes may present a protective mechanism to maintain the tendon integrity in response to the increased demand due to the changed muscular strength.

As for most previous investigations on MTU structure and function in adult athletes (Kongsgaard et al., 2005; Arampatzis et al., 2007b; Stafilidis and Arampatzis, 2007; Wang et al., 2012; Couppé et al., 2013; Epro et al., 2019), the above findings rely on a single time point (data were assessed directly prior to the competition period) and therefore potential fluctuations in muscle strength and tendon stiffness over a period of athletic training is unknown. As part of this work on muscle and tendon adaptation, we investigated for the first time MTU biomechanical properties at about three- to five-week intervals over several years of athletic training and competition. When all data points analyzed within the 12-month observation period were taken into consideration (for the 18 healthy athletes monitored), we found greater fluctuations of muscle strength over time in elite jumpers in comparison to controls irrespective of the analyzed leg (i.e., greater residuals). Furthermore, the take-off leg showed statistically significant lower uniformity (lower CS) in adaptive changes of muscle strength in comparison to the control group although statistical significance was not achieved for the inter-group comparison of fluctuations in tendon stiffness (*P* = 0.074) and maximal tendon strain (*P* = 0.092). There is therefore evidence that the notable fluctuations of muscle strength in the athlete group were only partly matched by an adaptive response in the tendon.

**Figure 7:**
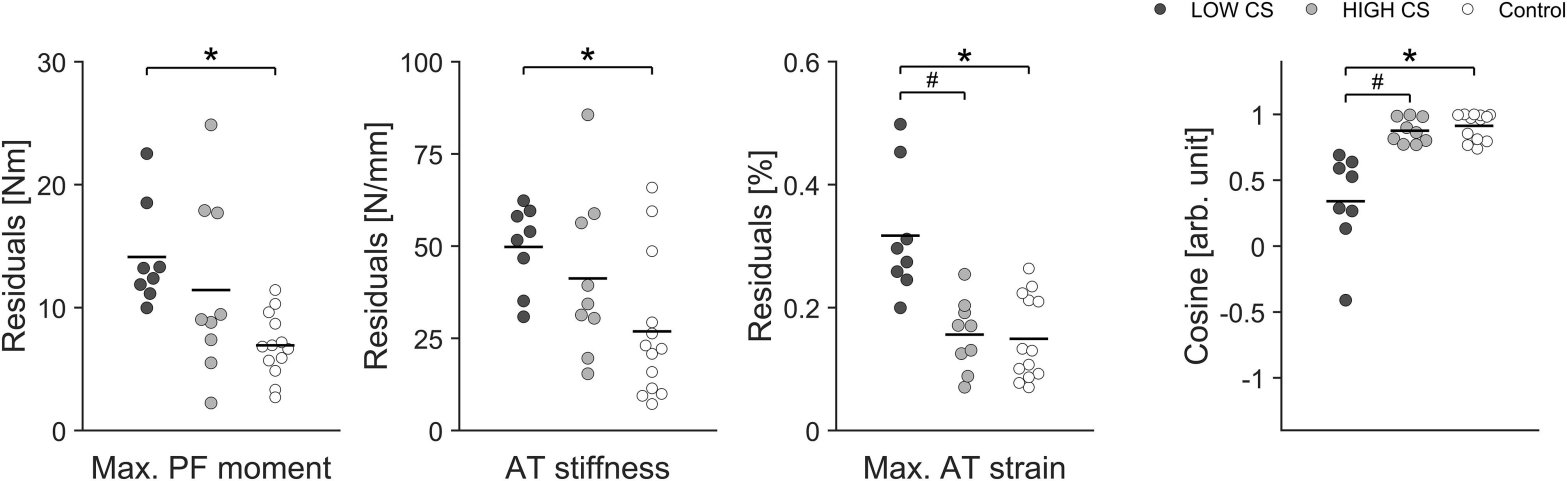
Individual residuals and group means (dark line) in muscle strength (Max. PF moment), tendon stiffness (AT stiffness) and maximal tendon strain (Max. AT strain) as well as the cosine similarity (Cosine) of the relative changes of muscle strength and tendon stiffness between sessions over the 1 year observation in the take-off leg of the Low CS and High CS group of elite jumpers and in the control group. *Statistically significant difference between Low CS and control group (*P* < 0.05). ^#^Statistically significant difference between Low CS and High CS group (*P* < 0.05).

A more detailed examination of our data, moreover, revealed that some elite athletes clearly showed higher fluctuations over time in maximal tendon strain especially in their take-off leg than other athletes and controls (see interquartile range and maximal value in Figure 2). Accordingly we decided in addition to use an approach based on median split for CS for the take-off leg of the elite athletes and divided them into two groups: Low CS (*n* = 8) and High CS (*n* = 9) athletes (see Figure 7). Using this approach we found statistically significant greater fluctuations of muscle strength (*P* = 0.007) and tendon stiffness (*P* = 0.032) for Low CS elite jumpers in comparison to controls (Figure 7). As a consequence, tendon strain level during maximal contractions recorded over time showed in athletes significantly greater fluctuations as well (*P* < 0.001; Figure 7); there were episodes for which levels of strain were up to 1.5 times higher in comparison to controls. Given that tendon strain is accepted as the main mechanical parameter determining tendon damage accumulation (Wren et al., 2003; Andarawis-Puri et al., 2012), this implies to a temporarily increased mechanical demand on the tendon over a season and hence a greater threat to the tissue integrity. Moreover, the findings from our additional two elite athletes with chronic AT tendinopathy confirmed previous reports of a decreased tendon stiffness and increased tendon strain in patients with tendinopathy (Wang et al., 2012; Kulig et al., 2016). The above findings, when combined with the relatively high prevalence (up to 40%) of AT overuse in elite sprinters and jumpers (Janssen et al., 2018) support the notion that unbalanced adaptation of muscle and tendon in elite jumpers may have a clinical relevance and increase the risk for overuse injuries.

Given that fluctuation of muscle strength was more predominant than in tendon stiffness, we suggest that the fluctuation of strain was primarily due to the variations in muscle strength rather than tendon stiffness. Taking into account that tendons have a lower tissue turnover than muscles (Heinemeier et al., 2013) we argue that muscular adaptation takes place at a higher rate in response to modified mechanical loading induced by variations in training volume and content over a season. Regarding this, it is, however, important to note that the six elite athletes that we monitored over 4 years showed no indications for different temporal dynamics in muscle and tendon adaptation between the preparation and competition periods (Figure 4). These athletes demonstrated no significant differences in fluctuations of muscle strength and tendon stiffness and hence maximal tendon strain between preparation and competition periods, irrespective of the leg analyzed. Therefore, while we argue that the above observed non-uniform adaptation in elite athletes could be defined by the differences in the MTU temporal dynamics, potential variations in the training stimulus between preparation and competition were insufficient to cause measurable differences in the adaptive changes within the MTU in elite jumpers.

Adaptation of the MTU to various mechanical alternations has been the subject of numerous studies (Bohm et al., 2015a) and has provided evidence that the muscle and tendon homeostatic response to mechanical loading can vary considerably. For example, a discrepancy between muscle and tendon in the time course of adaptation in response to mechanical loading has been reported previously for both the patellar and the Achilles tendon (Kubo et al., 2012), revealing that muscle strength gains can precede significant changes in tendon biomechanical properties. While an increase in tendon stiffness relies on modulation of tissue metabolism and subsequent adaptive changes of tendon structure and morphology (Kjær et al., 2009), neuronal adaptation enables marked increases in muscle strength even before major morphological changes occur within a muscle (Moritani and DeVries, 1979; Narici et al., 1989). Moreover, there is strong evidence that muscles tend to have superior plasticity in response to a wide range of exercise modalities in comparison to tendons, as both high and low loading magnitudes can facilitate increases in muscle strength but only high magnitude loading promotes tendon adaptation (Arampatzis et al., 2007a). Therefore, in line with recent studies conducted on the patellar tendons of adolescent volleyball players (Mersmann et al., 2016, 2017), we propose that injury prevention strategies in elite track and field athletes might need to integrate mechanical stimuli which primarily focuses on effectively increasing the tolerance of the tendon to mechanical loading i.e., by incorporating exercise modalities involving optimal strain magnitudes over longer strain durations (Arampatzis et al., 2007a; Bohm et al., 2014).

Interestingly, the individual tendon strain in the take-off leg showed greater fluctuations over 1 year of athletic training and competition for most but not for all elite athletes in comparison to controls (Figure 7). Moreover, the differences to control subjects were in absolute terms obvious for the swing leg but did not reach statistical significance (Figure 2). This substantial variation amongst elite athletes indicates that the relative time courses of adaptation in muscle and tendon might reveal differences at an individual level and that imbalances between muscle and tendon could often remain undetected when only group mean values are considered. Therefore we argue that individualized approaches (Arampatzis et al., 2020) and rapid subject-specific tendon strain estimates (Pizzolato et al., 2020) are needed to provide valuable information for coaches and athletes as well as their medical teams about the adaptive processes in muscles and tendons during the various phases of athletic training. This would enable detection of temporal disruption of the fine-tuned interaction within the MTU during the course of training and could allow adjustment of individual athlete training loads through tailored intervention.

With regard to our case study reporting of two elite athletes with unilateral chronic AT tendinopathy, we saw no evidence for differences in contractile strength between limbs but there was lower tendon stiffness in the affected limb. As a consequence, increased tendon strain during maximal contraction was identified for the affected limb, in accordance with previous results for both non-active adults and for athletes with tendinopathy (Wang et al., 2012; Kulig et al., 2016). Moreover, we were able to expand those findings, showing that AT tendinopathy is accompanied by lower uniformity (lower CS) in the adaptation changes of muscle strength and tendon stiffness, leading to higher fluctuations in tendon strain over time. This further supports the notion that an unbalanced adaptation within the MTU in athletes could heighten the risk for overuse injuries. In contrast to this, the case report data of one elite jumping athlete 10 months after AT rupture and reconstruction indicated opposing findings: there was a lower force generation capacity but a higher tendon stiffness in the affected limb, leading to a lower level of strain. These bilateral differences and deficient contractile strength in the affected limb seemed persistent as the athlete did not show any adaptive improvements over a follow up period of 2.5 years post-surgery (Figure 6). Similar findings have been obtained in several cross-sectional and longitudinal investigations of non-active AT rupture patients even more than 10 years post rupture and reconstruction (Agres et al., 2015; Heikkinen et al., 2017). As the current longitudinal data were generated from an elite athlete encountering athletics training with high training loads and volumes over a period of 2.5 years post-surgery we may argue that AT rupture and reconstruction lead to irreversible MTU dysfunction. In contrast to an injury or pathology, an unbalanced adaptation of muscle and tendon due to athletic training in elite jumpers seems to be a temporary phenomenon. Further long-term investigations are needed to determine whether targeting balanced development of muscle strength and tendon stiffness through individualized MTU-focused exercise interventions can enhance the effectiveness of tissue recovery caused by injury or prevent overuse tendon injuries in athletes.

In relation to our testing protocol one could argue that the used 90° knee joint configuration could place the m. gastrocnemius medialis to an unfavorable position to generate force based on its force-length relationship, which might lead to a difference in tendon subcomponent deformations and affect the determined tendon stiffness. In justification, the primary reason for using the flexed knee joint angle configuration was to minimize the inevitable ankle joint rotation that occurs when using an extended knee joint, as this causes a substantial overestimation of the tendon elongation due to the generated force during the plantar flexion contractions. In an earlier study (Ackermans et al., 2016) we investigated the current (90°) and a more dorsiflexed ankle joint configuration (85°) with the same knee joint setup to enhance the gastrocnemii force potential and their contribution to the resultant joint moment. Even though the more dorsiflexed position demonstrated significantly higher ankle joint moments and tendon elongation, no significant differences were found for tendon stiffness between those two joint configurations (Ackermans et al., 2016; Supplementary Material of the current study). Hence, although a flexed knee joint could potentially influence the interactions between the individual AT subtendons, it seems not to cause a clear measurable alteration in the m. gastrocnemius medialis tendon force-elongation relationship during loading, particularly in the “linear” region were the tendon stiffness was determined. However, we cannot exclude that the observed higher tendon strain fluctuations in elite athletes may be influenced also by variations in the regional strain patterns and subtendon sliding (Handsfield et al., 2020; Maas et al., 2020). Incorporating regional and subtendon strain differentiation could contribute additional understanding into the complex tendon loading pattern during various contractions in elite athletes. Further, the current work made use of generic AT moment arms from previous literature (Maganaris et al., 1998), which might affect the calculated tendon stiffness in absolute terms. However, we believe this could not affect the main outcomes from our longitudinal analyses and the non-uniformity of muscle and tendon adaptations in mature elite athletes. Finally, it is important to note that only six elite athletes were considered in our comparison between preparation and competition periods, irrespective of our monitoring both limbs over a period of 4 years of athletic training. Further studies are needed to investigate whether potential variations of training volume and content between preparation and competition can lead to amplification of non-uniformity in adaptation of muscle and tendon. This may be of particular relevance as it has been reported that the frequency of musculoskeletal injuries occurring during preparation is threefold greater in comparison to competition in elite track and field athletes (D'Souza, 1994).

## Conclusions

In conclusion, the current findings demonstrate that repetitive plyometric and habitual mechanical loading during training and competition can affect uniformity within the MTU in elite jumpers, leading to a temporarily increased demand on the tendon and potentially higher injury risk. We argue that the mechanism for unbalanced MTU adaptation during athletic training is related to differences in the time courses of muscle and tendon adaptation, which needs to be considered in exercise and rehabilitation. Further long-term investigations should address the potential of more individualized exercise interventions for prevention of disruptions to the fine-tuned interactions within the MTU in athletes.

## Data Availability Statement

The raw data supporting the conclusions of this article will be made available by the authors, without undue reservation.

## Ethics Statement

The studies involving human participants were reviewed and approved by German Sport University Cologne Ethics Committee. The patients/participants provided their written informed consent to participate in this study.

## Author Contributions

KK and GE conception and design of research, performed experiments, analyzed data, interpreted results of experiments, prepared figures, drafted manuscript and approved final version of manuscript.

## Conflict of Interest

KK has equity in Protendon GmbH & Co. KG, whose measurement device and software was used for the data processing and analysis in this study. The remaining author declares that the research was conducted in the absence of any commercial or financial relationships that could be construed as a potential conflict of interest.
