# Supplementary material for: Monitoring Muscle-Tendon Adaptation Over Several Years of Athletic Training and Competition in Elite Track and Field Jumpers
Source: Front Physiol. 2020 Dec 16;11:607544. doi: 10.3389/fphys.2020.607544 (PMC7772406; doi:10.3389/fphys.2020.607544)
Supplement: Supplementary file 1 [file Data_Sheet_1.DOCX]

Supplementary Material

Monitoring muscle-tendon adaptation over several years of athletic training and competition in elite track and field jumpers

Kiros Karamanidis^1*^, Gaspar Epro^1^

^1^Sport and Exercise Science Research Centre, School of Applied Sciences, London South Bank University, London, United Kingdom

*** Correspondence:**Kiros Karamanidis
k.karamanidis@lsbu.ac.uk

**Table 1**: Description of the analysed elite track and field jumpers including the personal best results.

|  | **High jump** | **Triple jump** | **Long jump** | **Pole vault** |
| --- | --- | --- | --- | --- |
| Subjects (n) | 11 M  11 F | 6 M  5 F | 9 M  8 F | 9 M  8 F |
| Age (years) | 24 ± 5  23 ± 3 | 22 ± 3  23 ± 4 | 22 ± 3  24 ± 4 | 22 ± 4  25 ± 6 |
| Body height (cm) | 195 ± 4  182 ± 6 | 187 ± 8  177 ± 5 | 185 ± 6  177 ± 5 | 187 ± 4  172 ± 8 |
| Body mass (kg) | 82 ± 7  65 ± 5 | 87 ± 7  60 ± 5 | 80 ± 9  64 ± 7 | 81 ± 6  62 ± 7 |
| BMI (kg/m^2^) | 21.6 ± 1.8  19.7 ± 1.1 | 24.8 ± 3.6  19.2 ± 0.6 | 23.2 ± 2.0  20.4 ± 1.7 | 23.2 ± 1.3  21.0 ± 1.4 |
| Personal best result (m) | 2.27 ± 0.05  1.90 ± 0.05 | 16.43 ± 0.60  13.81 ± 0.76 | 8.01 ± 0.26  6.72 ± 0.20 | 5.66 ± 0.21  4.58 ± 0.22 |

Values are expressed as means ± SD. M, male; F, female; BMI, body mass index.

**Methodological considerations and pilot data**

Prior performing the current study we carried out several pilot studies to test the accuracy of the implemented method to assess the triceps surae (TS) muscle-tendon unit (MTU) mechanical properties. Even though the used 90 degree knee joint configuration allows a more secure fixation of the limb, and hence result in a much lower angular rotation at the ankle joint in comparison to straight knee positioning (on average inevitable ankle joint changes of about 13-14 degrees when using a fully extended knee joint angle during maximal isometric voluntary plantarflexion contractions; see e.g.: Arampatzis et al., 2005; Karamanidis et al., 2005), this could place the *m. gastrocnemius medialis* (GM) into a less favorable position to generate force based on the force-length relationship. Accordingly, this could potentially influence, next to the maximal tendon strain, also the assessed length-tension relationship of the tendon. In order to address this drawback we performed a pilot study setting the ankle joint into a more dorsiflexed position (85° ankle joint angle). By doing so, the entire TS MTU was in a more lengthened position, placing more tension on the GM tendon (less slack) and leading to a rightwards shift in the force-length relationship of the *gastrocnemii* muscles. Hence, increasing the GM force potential and contribution to the net ankle joint moment (Arampatzis et al., 2006). In this pilot study with 10 young healthy adults, we found for the 85° in comparison to the 90° ankle joint position a significantly (P < 0.05) higher ankle joint moment (mean and SD; 322 ± 46 N·m vs. 277 ± 35 N·m) and maximal tendon elongation (13.6 ± 2.8 mm vs. 12.3 ± 2.3 mm; Fig 1). However, no significant difference was detected in tendon stiffness between the two analysed ankle joint configurations (1219 ± 461 N·mm^-1^ vs. 1210 ± 283 N·mm^-1^; Fig 1). Thus, although the force generation capacity of the *triceps surae* muscle is reduced in a more shortened MTU length, decreasing total lengthening of the tendon during maximal isometric voluntary contractions, it appears not to cause clear measurable effects on the force-elongation relationship of the tendon during the loading phase.

**
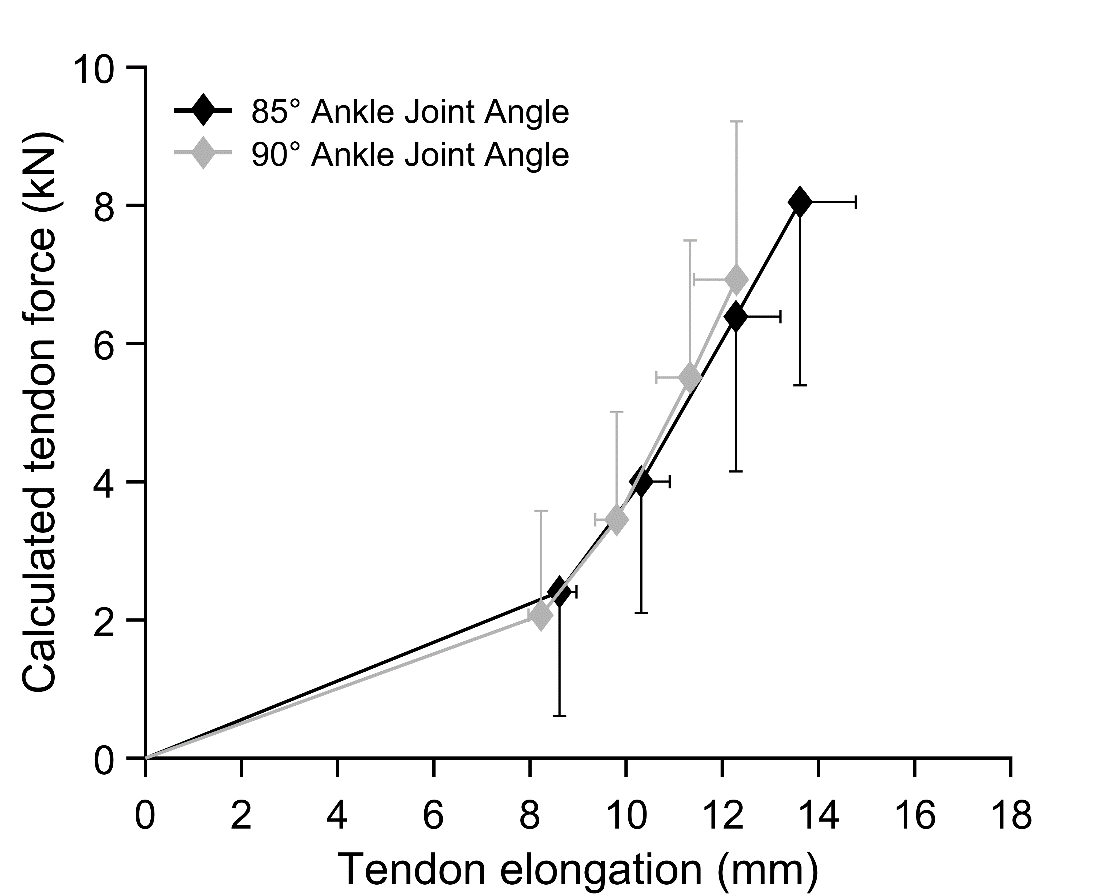
**

**Fig. 1**: The force-elongation relationship (mean and SD) of the *m. gastrocnemius medialis* tendon in 10 healthy young adults, assessed either using an ankle joint angle 90° (foot perpendicular to the shank) or a more dorsiflexed ankle joint angle 85°, with the knee joint set at an 90° angle (thigh perpendicular to shank).

In a second pilot study with nine young healthy adults, we investigated different calculation methods for the estimated inevitable ankle joint angular changes during isometric contractions in the same seated position (90° angle at the ankle and knee joints) as in the current study. In this pilot study we aimed to compare the method of estimating ankle joint angular changes using the vertical displacement of a potentiometer located under the heel (current method) with the sagittal plane (2D kinematic analysis) of ankle joint angular changes assessed by tracking reflective markers of the lower extremity using a motion capture system (120Hz, Qualisys, Gothenburg, Sweden). The potentiometer method was based on the heel elevation during contraction, by which the alterations in the ankle joint angle from resting state until maximal plantarflexion moment were calculated via the inverse tangent of the ratio of the heel lift to the distance between the head of the fifth metatarsal bone and the axis of the potentiometer (Fig. 2C). The pilot study found the current method to be in appropriate accordance with the 2D motion capture analysis, with the absolute difference lower than 1.1° in ankle joint angular changes during the maximal isometric plantarflexion contractions (from rest to the maximal ankle joint moment; current method: 3.8 ± 1.1°; 2D motion capture: 4.9 ± 1.4°).

**Fig. 2:** Experimental setup and methodology of the used custom-made strain-gauge type dynamometer (TEMULAB®,
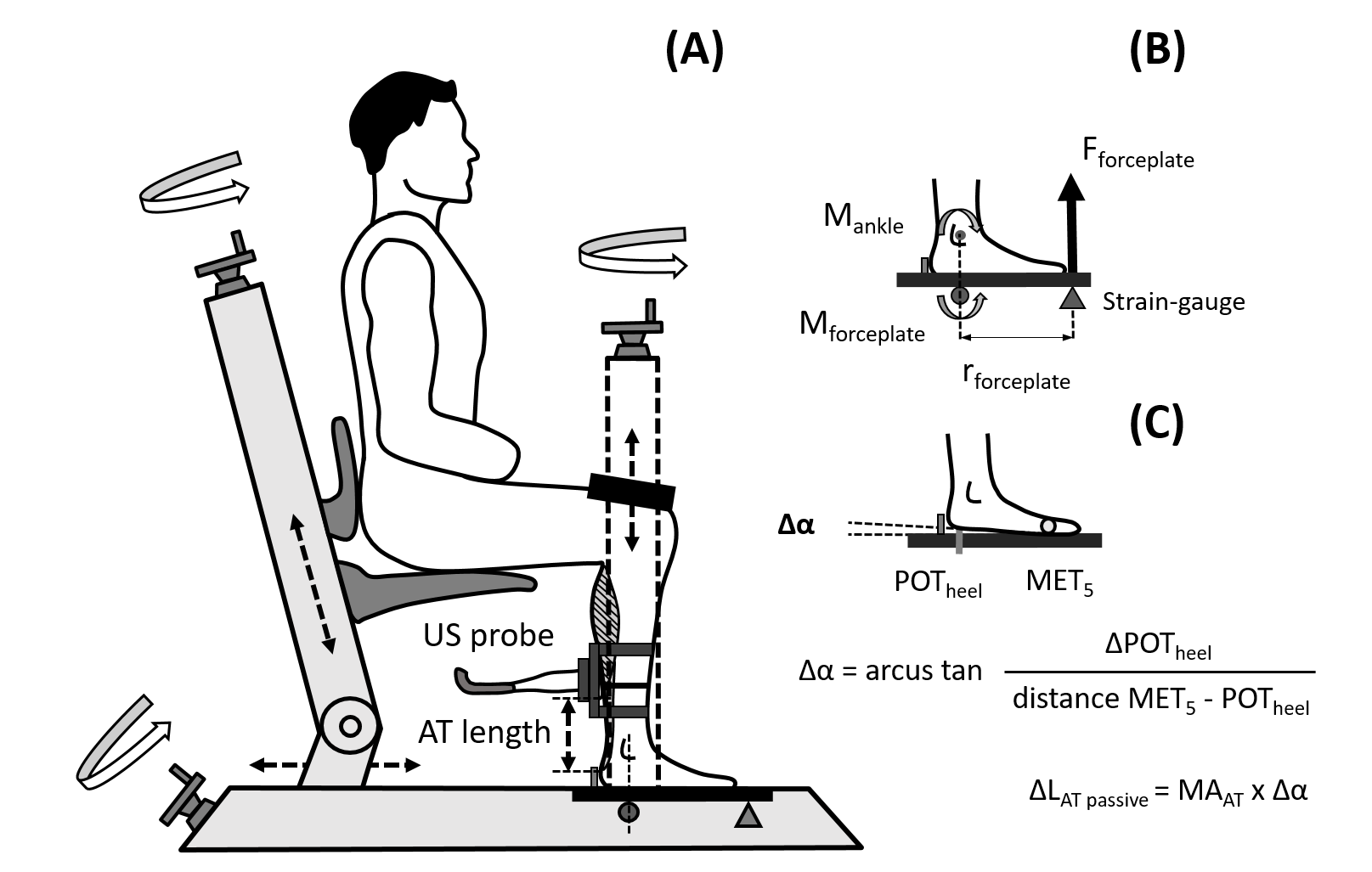
Protendon GmbH & Co. KG, Aachen, Germany). A: The participant is seated with the knee and ankle joints fixed at 90° angle (thigh and foot perpendicular to the shank) with the foot placed on a custom-made strain-gauge type dynamometer (1000 Hz). An integrated laser-guided potentiometer system was used to position the foot on the dynamometer as well as to measure the tendon’s resting length (AT length) between the tuber calcanei and myotendinous junction (MTJ) of m. gastrocnemius medialis (Ackermans et al., 2016). The ultrasound probe (US probe) was securely fixed on the shank in a custom-made case using adjustable straps parallel to the tibia just above the MTJ. B: The axis of rotation of the ankle was aligned with the force plate’s center of rotation to equalize the measured (M_forceplate_) and generated moment (M_ankle_), which is the product of F_forceplate_ (resultant force of the force plate) and r_forceplate_ (moment arm of the force plate). C: The effect of potential ankle joint rotation on the measured tendon elongation during each contraction was accounted for by subtracting the estimated elongation due to ankle joint changes (ΔL_AT passive_), the product of a constant moment arm (MA_AT_) acquired for the literature (Maganaris et al. 1998) and the ankle joint changes (Δα), which was calculated via an inverse tangent of the ratio of heel lift (measured via a heel-potentiometer; POT_heel_) and ankle joint axis distance to the head of the fifth metatarsal bone (MET_5_) as in Ackermans et al. (2016).

With regard to the assessment of the ankle joint changes during contraction, to assess tendon elongation due to the generated force and account for the effect of ankle joint rotation on tendon elongation, we used a constant moment arm (MA_AT_) provided in the literature (Maganaris et al. 1998) and estimated the displacement of the tendon origin (ΔL_AT passive_) caused by the ankle joint angular changes (Δα) via the product of Δα (in rad) and the constant MA (Fig 2). This method is based on the “tendon travel method” equation proposed by An et al. (1984), which is often used in the literature to assess AT moment arms by ultrasonography *in vivo*. It should be noted that in the current pilot study, heel lift was relatively low and hence our calculated Δα was, on average, 3.8 degrees (this value was slightly higher than the Δα in the current manuscript as we used a slightly different rigid frame in the pilot testing) and thereby clearly lower than in previous studies (with fully-extended knee joint) due to the experimental setup and our rigid dynamometer. However, assuming that the MA_AT_ taken from the literature has an error of 1 cm, the error in ΔL_AT passive_ at maximal Δα (3.8° = ~0.0663 rad) would be: ΔL_AT passive_ = 0.0663 * 0.01 = 0.000663 m. Please also note that there were no significant differences in heel rise (POT_heel_ - assessed by the potentiometer reading), and hence in Δα, between measurement sessions in our longitudinal analysis, meaning that a potential error in ΔL_AT passive_ (caused by errors in MA_AT_) did not change between measurement sessions. Furthermore, in the current study the resultant ankle joint moments were assessed using only the vertical component of the ground reaction force (GRF), thereby neglecting the directional changes of the force vector during the contraction. In order to compare the maximal ankle joint moments calculated solely using by the vertical component of the GRF with the moments calculated using all three dimensions of the GRF, in the above described pilot study with 9 subjects we obtained simultaneously also the 3D GRFs during the maximal isometric plantarflexion contractions using, next to the motion capture system, a force plate (1080 Hz, 400 x 600 mm, Bertec, Columbus OH, USA). The findings demonstrated that the maximal ankle joint moment assessed using only the vertical component was on average 8% greater in comparison to the 3D calculations (157.8 ± 47.7 N·m *vs.* 146.3 ± 40.9 N·m). Nevertheless, a significant correlation was found between the two methods of calculating maximal ankle joint moment (R^2^ = 0.996, P < 0.01; Fig. 3), hence demonstrating that the relative difference between methods is constant (systematic error), and that using only the vertical component of the GRF for calculating the ankle joint moments during isometric contractions is an appropriate approach for the current investigation. In addition, in this pilot study we examined also the effect of ankle joint-dynamometer axis misalignment on the estimated joint moments, by investigating the maximal anterior displacement of the ankle joint axis during maximal isometric plantarflexion contractions. The maximal anterior shift of the ankle joint axis during the maximal isometric plantarflexion contractions was on average 3.4 ± 2.1 mm, which thereby can lead to an overestimation of calculated joint moments. However, when relating this anterior shift to the moment arm of the GRF acting about the ankle joint, the relative change was as low as ~1.7%. Accordingly, this drawback can have merely a negligible effect on our joint moment calculations.

**
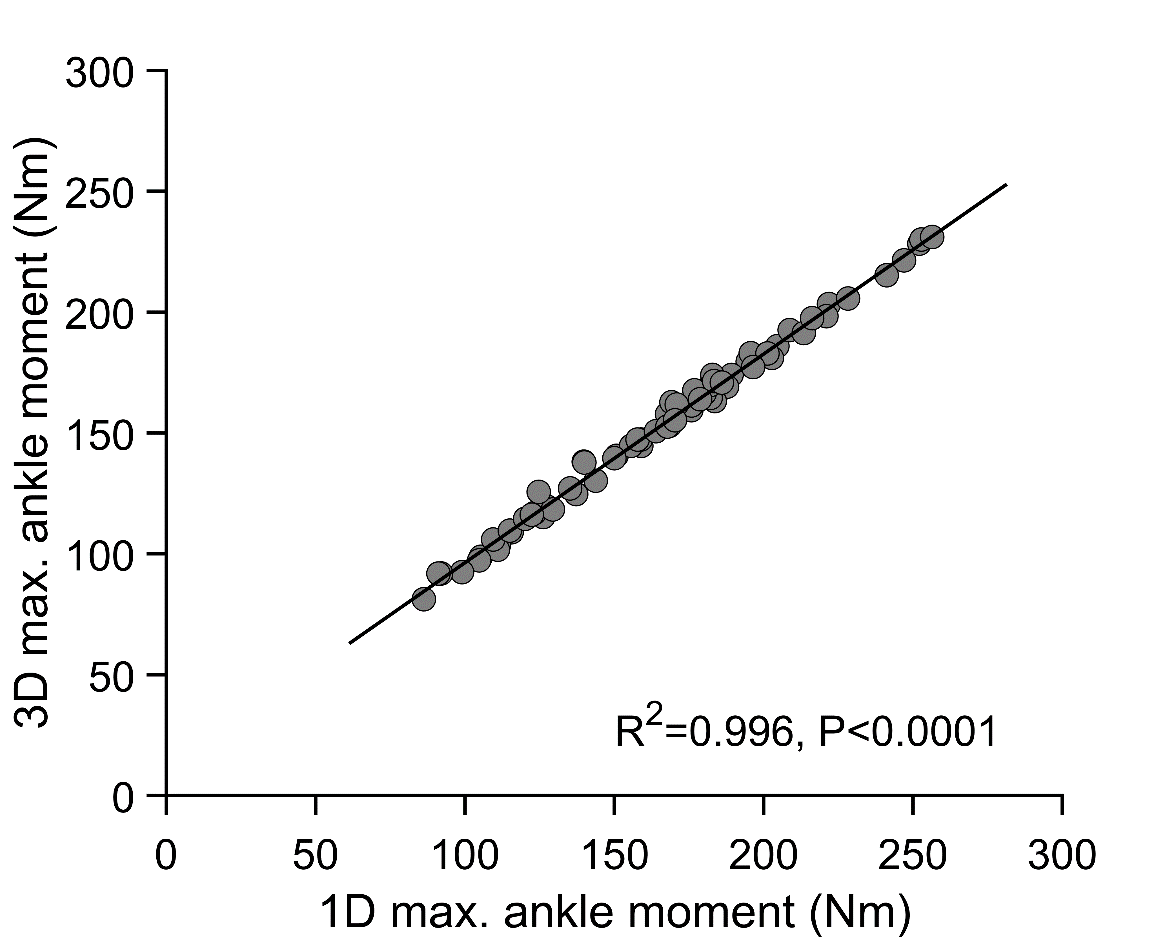
**

**Fig. 3:** Relationship between the maximal ankle joint moments during an isometric voluntary plantarflexion contraction estimated by using only the vertical component of the ground reaction force (1D max. ankle moment) and by considering all three components of the ground reaction force (3D max. ankle moment).

Furthermore, one might argue against calculating the secant stiffness and not the tangential stiffness of the tendon due to potential measurement errors. In the current study, the tendon stiffness was calculated as the ratio between the change in force and elongation from 30% to 80% of MVC, hence using merely two data points to determine the tendon stiffness. Nevertheless, by using the sustained contractions at the target joint moments leads to a clear steady state on the ultrasound image sequence, allowing an easier digitalization process than during dynamic contractions where the tendon elongates rather rapidly during MVC, which is particularly problematic at lower ultrasound sampling frequencies (< 30 Hz). Using sustained contractions may be better to exclude potential measurement errors due to a low ultrasound sampling frequency or potential time delays of synchronization between the ultrasound and dynamometer data during ramp contractions (see e.g.: Finni et al. 2013; Seynnes et al. 2015). In addition, the theoretical consideration behind using the sustained method was to negate loading rate dependency of the tendon as it allows to take into account any possible phase shift (due to tendon’s time-dependent viscous properties) of the reactive behaviour of viscoelastic material (Meyers and Chawla, 1999). This can be described via a simple Kelvin-Voigt model, which consists of parallel connected purely viscous damper and purely elastic spring (see examples of applying the Kelvin-Voigt model in biological tissue assessments: Alkalay et al., 2015; Kiss et al., 2004; Tzschätzsch et al., 2014). Based on this model, external stress causes the spring to deform while damper acts against deformation, causing thereby a time delay in the deformation. Regarding this issue, in a recent study (McCrum et al., 2018), we were able to detect loading rate effects on tendon stiffness only up to 25% of the MVC plantar flexion contractions, which were reduced by using the sustained method.

Next to the method-comparisons described above, regarding the reliability of the measurements, in one of our previous studies (Ackermans et al. 2016) we found an average relative difference in TS MTU mechanical properties between two consecutive measurement days of 0.9% (muscle strength) and 3.1% (tendon stiffness), with statistically significant (P < 0.05) ICC values of 0.96 and 0.64 for the maximal plantarflexion moment and tendon stiffness, respectively (Fig. 4). Since the average relative differences in MTU properties between elite athletes and controls in the current study were reasonably high (15-20%), we can conclude that the current approach was valid to detect such group related difference. Moreover, as we used the same experiment setup and procedure for our within- and between subject group comparison and because residuals in maximal tendon strain were two times higher for the elite athletes (low CS) in comparison to the group control, we are confident that our method was sensitive **
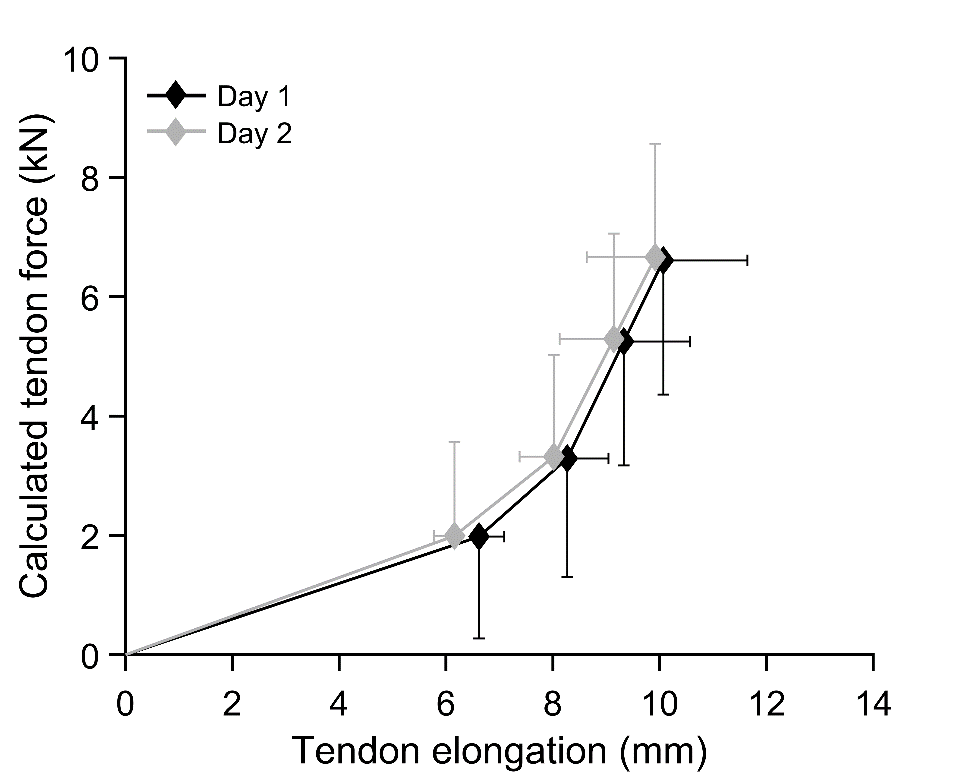
**enough to detect the reported differences.

**Fig. 4:** The force-elongation relationship (mean and SD) of the m. gastrocnemius medialis tendon in two consecutive days (Day 1 and Day 2) in 10 healthy young adults.

**References (Supplementary Material)**

Ackermans TMA, Epro G, McCrum C, Oberländer KD, Suhr F, Drost MR, Meijer K, Karamanidis K (2016). Aging and the effects of a half marathon on Achilles tendon force–elongation relationship. Eur J Appl Physiol 116: 2281–2292.

Alkalay RN, Vader D, Hackney D (2015). The degenerative state of the intervertebral disk independently predicts the failure of human lumbar spine to high rate loading: an experimental study. Clin Biomech 30: 211–218.

An KN, Takahashi K, Harrigan TP, Chao EY (1984). Determination of muscle orientations and moment arms. J Biomech Eng 106: 280-282.

Arampatzis A, Stafilidis S, DeMonte G, Karamanidis K, Morey-Klapsing G, Brüggemann GP (2005) Strain and elongation of the human gastrocnemius tendon and aponeurosis during maximal plantarflexion effort. J Biomech 38: 833-841.

Arampatzis A, Karamanidis K, Stafilidis S, Morey-Klapsing G, DeMonte G, Brüggemann GP (2006) Effect of different ankle-and knee-joint positions on gastrocnemius medialis fascicle length and EMG activity during isometric plantar flexion. J Biomech 39: 1891-1902.

Finni T, Peltonen J, Stenroth L, Cronin NJ (2013). Viewpoint: on the hysteresis in the human Achilles tendon. J Appl Physiol 114: 515–517.

Karamanidis K, Stafilidis S, DeMonte G, Morey-Klapsing G, Brüggemann GP, Arampatzis A (2005) Inevitable joint angular rotation affects muscle architecture during isometric contraction. J Electromyogr Kines 15: 608-16.

Kiss MZ, Varghese T, Hall TJ (2004). Viscoelastic characterization of in vitro canine tissue. Phys Med Biol 49: 4207–4218.

Maganaris CN, Baltzopoulos V, Sargeant AJ (1998). Changes in Achilles tendon moment arm from rest to maximum isometric plantarflexion: in vivo observations in man. J Physiol 510: 977-985.

McCrum C, Oberländer KD, Epro G, Krauss P, James DC, Reeves ND, Karamanidis K (2018). Loading rate and contraction duration effects on in vivo human Achilles tendon mechanical properties. Clin Physiol Funct Imaging 38: 517–523.

Meyers AM, Chawla KK. Mechanical Behavior of Materials (1999). Prentice Hall, Upper Saddle

River, NJ.

Seynnes OR, Bojsen-Møller J, Albracht K, Arndt A, Cronin NJ, Finni T, Magnusson SP (2015). Ultrasound-based testing of tendon mechanical properties: a critical evaluation. J Appl Physiol 118: 133–141.

Tzschätzsch H, Ipek-Ugay S, Guo J, Streitberger K-J, Gentz E, Fischer T, Klaua R, Schultz M, Braun J, Sack I (2014). In vivo time-harmonic multifrequency elastography of the human liver. Phys Med Biol 59: 1641–1654.
